# Scale matters: a survey of the concepts of scale used in spatial disciplines

**DOI:** 10.1080/22797254.2019.1626291

**Published:** 2019-06-10

**Authors:** Zahra Dabiri, Thomas Blaschke

**Affiliations:** Department of Geoinformatics-Z_GIS, University of Salzburg, Salzburg, Austria

**Keywords:** Spatial scale, scale-space theory, cartographic scale, geographic scale, operational scale, observation scale, measurement scale, modelling scale, policy scale

## Abstract

Scale is a critical factor when studying patterns and the processes that cause them. A variety of approaches have been used to define the concept of scale but confusion and ambiguities remain regarding scale types and their definitions. The objectives of this study were therefore (1) to review existing types and definitions of scale, and (2) to systematically investigate the ambiguities in scale definitions and to determine the applicability of the various scale types and definitions. Through a comprehensive literature review, we identified seven types of scales and designed a survey for the seven definitions of scale and interviewed 150 scientists. The results show that the more cartography related types of scale are relatively well known while the more abstract dimensions are less known and are most ambiguous. Based on graphical examples, participants were asked which spatial scales are most relevant for their work. Surprisingly, composite objects such as a forest stand were most relevant followed by individual objects such as single trees and, lastly, more generalized categorizes or meta-objects such as “forested area”. We have drawn some conclusions that will help to clarify the different types of scale in regard to their practical use.

## Introduction

The term scale has many meanings, and it is strongly dependent on context (Goodchild, 2001a). For instance, in human-geography, the term scale has been reckoned to have three aspects, namely size (e.g. census tract, province, continent), level (e.g. local, regional, national), and relation (as a complex mix that includes space, place and environment)(Howitt, 1998). In the context of digital technology, Goodchild (2001b) categorized four meanings for the term scale: (1) implication of level of spatial detail, (2) representative fraction, (3) spatial extent and (4) process scale. In the context of remote sensing, previous studies emphasized that for the objects and phenomena in the real world, is their inherent existence over certain ranges of scale (Koenderink, 1984; Lindeberg & Ter Haar Romeny, 1994). Objects appear in different ways depending on the scale of observation. An evident example to clarify the object scale-dependency is the concept of a branch of a tree, which is meaningful to discuss it at a scale of a centimetre to maximum meter, and it is meaningless to discuss it at the scale of a nanometre or a kilometre. At the latter scales, it is more relevant to talk about the molecules that form the leaves of the tree or the forest in which the tree grows (Lindeberg, 1994).

In geoscience disciplines (also known as Earth-sciences), scale concepts are well recognized as a critical factor when studying patterns in nature and the processes that cause them. A number of different types of scale have been identified in an attempt to improve the reliability of outputs from spatial planning models (Bierkens, Finke, & De Willigen., 2000; Gibson, Ostrom, & Ahn, 2000; Peterson & Thomas Parker, 1998; Sheppard & McMaster, 2008; Withers & Meentemeyer, 1999; Wu & Qi., 2000). Nevertheless, a degree of confusion and ambiguity remains in a number of definitions of scale used in different scientific fields, for example, in research dealing with spatial heterogeneity, spatial patterns and their underlying processes, changes in physical entities, and population distributions, to mention just a few examples (Gibson et al., 2000; Marceau, 1999; Steele, 1991; Turner, 1989; Wiens, 1989). The ambiguities in definitions of scale relate mainly to confused general concepts and to the absence of an accepted, uniform terminology (Quattrochi & Goodchild, 1997; Sheppard & McMaster, 2008; Turner, 1989; Withers & Meentemeyer, 1999; Wu, Jones, Li, & Loucks, 2006).

The objectives of this study were therefore (1) to review the existing types, and definitions of scale in geoscience disciplines, and (2) to demonstrate the ambiguities in definitions of scale, and to determine the applicability of the various definitions. To achieve these objectives a survey was designed based on data collected from a comprehensive literature review and interviews with 150 scientific researchers from a variety of geospatial disciplines, and three major groups including (1) PhD candidates, (2) researchers and (3) graduate and undergraduate students. We decided to approach these ambiguities in scale definitions and the absence of an accepted, uniform terminology with an empirical survey which shall find out what terms users are really familiar with and what level of detail in terms of single objects, groups of objects and composite elements they are aiming for. To our best knowledge, this is the first international systematic survey of scale types and definitions. The reminder of the article is structured as follows: section 2 presents detailed descriptions of the provided scale definitions from different geoscience disciplines. Section 3 introduces the design of the survey. Section 4 describes the results of the survey. Section 5 and 6 provide a discussion and conclusions on how to interpret the results of the survey.

## The definitions of scale and the importance of scale in geoscience disciplines

### Definitions of scale

Koenderink (1984) emphasized that in any image situation we have to face the problem of scale; a given image has a limited extent or window (i.e. o*uter scale*) and limited resolution (i.e. *inner scale*). In the real world, the *outer scale* of an object or a feature is a (minimum) size of a window that completely contains the object or the feature. The *inner scale* is corresponding to the scale at which the substructure of the object or the feature begins to appear. In remote sensing, the *inner scale* is limited by the resolution of the sampling device (grid or pixel size) and an *outer scale* limited by the field of view (Florack, Ter Haar Romeny, Koenderink, & Viergever, 1992). The concept of spatial resolution is closely related to scale (Cao, 1992). Spatial resolution is referred to the smallest distinguishable parts of an object observed by a remote sensing platform (Tobler, 1988), and the choice of the appropriate spatial scale (i.e. grid and field of view) for modelling a real-world “*inner*” and “*outer*” scale is limited to the sensors' specifications. The selection of the appropriate scale and resolution is multifaceted. Woodcock and Strahler (1987) analysed the spatial structure of the image using graph local variance, as an indication between the environment under investigation and spatial resolution.

According to Allen and Starr (1982, p. 18), the scale was defined as a period of time or space over which signals are integrated or smoothed to give a message. Therefore, the scale is determined by integration of signal over a window (time period) that moves continuously down the signals with time, applying equal weight (in a simple case) or not-constant (in usual cases) to all parts of the signal stream within the window. In this quote, the scale is a weighted integration of the signal, and it is determined as:

(1) $$S=\int_{-\infty }^{t}N\left(t^{\prime }\right)Q\left(t-t^{\prime },t\right)dt^{\prime }$$

where *N* is the signal, *Q* is the two-dimensional weighting function. In other words, the scale is an operation which implies filtering using a moving window.

Witkin (1983) ***considers scale as a continuous parameter, which is the generalizing of the existing notion of Gaussian pyramids. The relationship between diffusion equations and image structures between different scales was developed into scale-space theory (***Lindeberg, 1994; Lindeberg & Ter Haar Romeny, 1994***). Here, signals in an image are smoothed, using convolution of the image with filters, such as Gaussian derivatives of successively increasing width (t). 2.2 Definitions of scale in geoscience disciplines***

In geoscience disciplines such as geography, ecology, landscape ecology, or geoinformatics, and also for some scientific organizations, scale is a critical factor when studying physical processes, functions, and changes, including the study of human-environment interactions. This paper distinguishes between “Cartographic scale” and possibly “Modelling scales”, which are representative fraction and summary of the properties on the map and the data it represents (Goodchild, 2001b), and all the other types of scale, which are not ratios but simply measurements of magnitude (e.g. dimensions in time or space) – and hence refer to a completely different meaning of the word “scale”. Only one type of “Cartographic scale” exists, but there are an infinite number of possibilities when the word scale is used to refer to a magnitude rather than a ratio. Issues of scale related to map scale are fairly mechanical and can relatively easily be determined and, eventually, resolved (Fairclough, 2006).

In ecology, scale is known to be a fundamental concept when studying biodiversity patterns and the processes that cause them (Allen & Hoekstra, 2015; Allen & Starr, 2017; Elith & Leathwick, 2009; Foody & Curran, 1994; Gustafson, 2019; Holling, 2001; Kedron, Frazier, Ovando-Montejo, & Wang, 2018; Lam & Quattrochi, 1992; Levin, 1992; O’Neill, Johnson, & King, 1989; Pianka, 2011; Quattrochi & Goodchild, 1997; Robinson, 1950; Steele, 1991; Turner, Dale, & Gardner, 1989; Wiens, 1989; Woodcock & Strahler, 1987; Wu et al., 2006; Wu & Loucks, 1995). Ecologists defined spatial scale as having two main components: (1) grain, which is referring to the smallest spatial sampling units used to gather a series of observations, and (2) extent which is referring to the total area over which observations of a particular grain are made (O’Neill & King, 1998).

In landscape ecology, which addresses the complexities of ecological systems (Peterson & Thomas Parker, 1998), the scale is central to understanding heterogeneous spatial patterns (Levin, 1992; O’Neill et al., 1989; Tortini, Mayer, & Maianti, 2015; Turner et al., 1989). A natural scale (mostly used in natural sciences) is defined as a period of time and a spatial dimension at which ecological processes and physical characteristics occur within a landscape. In this case, the term “Natural scale” appears to overlap with the term “Operational scale”.

Geographic information system (GIS) and remote sensing are two main cores of Geoinformatics, which provide effective means for monitoring, detecting and measuring features for spatial analysis, thus helping to improve our understanding of such features (Foody & Curran, 1994; Goodchild, 2011; O’Neill et al., 1989; Quattrochi & Goodchild, 1997; Sheppard & McMaster, 2008; Turner, 1989; Turner et al., 2003; Woodcock, Strahler, & Jupp, 1988). With the development of remote sensing technology and the availability of different spatial, spectral, radiometric, and temporal resolutions, it is now possible to access near-real-time land surface information and to monitor changes in the environment more effectively, for example, by studying natural hazards at a number of different spatial and temporal scales (Benz, Hofmann, Willhauck, Lingenfelder, & Heynen, 2004; Hong, Adler, Negri, & Huffman, 2007; Klosterman et al., 2018; Maianti et al., 2014; O’Neill & King, 1998; Wan, Wang, & Xiaowen, 2004). In addition, three other attributes need to be taken into account in remote sensing, namely temporal, radiometric, and spectral scales (Warner, Foody, & Duane Nellis, 2009).

#### Summary of published definitions of scale in geoscience disciplines

Scale has two general dimensions: (1) the temporal scales, and (2) the spatial scales (Turner, 1989; Watt, 1947). Due to the importance of the spatial scales in understanding our environment, this article focuses mainly on spatial scales and their published definitions (for a comprehensive review the reader is referred to Blaschke (2006)). Wu and Zhao-Liang (2009) identified six spatial scale types (Table 1), whereby the main four were based on Lam and Quattrochi (1992), namely the observation scale, the operational scale, the geographic scale, and the cartographic scale. The other two types of scale mean policy scale and modelling scale were proposed by Bierkens et al. (2000).

**Table 1.** Different types of spatial scales in geoscience disciplines.

Important published definitions of different types of scale (and their sources)				
Types of scale	After Lam and Quattrochi (1992)	After Cao and Lam (1997)	After Sheppard and McMaster (2008)	After Wu & Zhao-Liang, 2009
Cartographic scale	The ratio between real world objects and their representations on maps, where a large-scale map covers smaller area but generally with more detail, and a small-scale map covers larger area with less detail	Also known as map scale: the proportion of a distance on a map to the corresponding distance on the ground	A map distance of a feature to that feature’s distance on the surface of the earth	The ration between distance on the map and on the ground
Geographic scale	Also known as observation scale: the spatial extent of a study area	Also known as observation scale: refers to the size or spatial extent of a study area	The spatial extent of a study area	The spatial extent of research
Operational scale	The spatial extent at which a particular phenomenon operates	The dimension of an operation at which certain processes operate in the natural environment	The dimension at which processes operate	The scale of action at which a certain process is supposed to operate
Observation (also known as measurement scale)	Not specified	Also known as spatial resolution: refers to the dimensions of the smallest distinguishable part of objects (such as pixels in remote sensing, or sampling intervals in an ecological study)	Also known as spatial resolution: details the granularity of the data set	The measurement units at which data is measured or sampled
Modelling scale	Not specified	Not specified	Not specified	The scale at which the model is built or derived
Policy scale	Not specified	Not specified	Not specified	The scale at which the decisions are made or the policy is implemented

According to Table 1 the following conclusions can be drawn with a high level of agreement:

- “Cartographic scale”: defined as a ratio between a distance on a map and its corresponding distance in the real world.
- “Geographic scale”: defined as the spatial extent of a research area.
- “Operational scale”: defined as the dimension over which a certain process operates.
- “Observation (or measurement) scale”: defined as the resolution or the size of the data set.

### Problems associated with scale definition

The broad types of spatial scales and their definitions, together with the lack of generally accepted scale definitions and terminology, may well lead to different interpretations between different disciplines and sub-disciplines (Weng, 2014). We assumed that despite the variety of approaches that have been used to define the concept of scale, confusion and ambiguities remain regarding these definitions. Therefore, a survey was designed and conducted for the empirical part of this study to establish the opinions of researchers on definitions of different types of scale. In order to prevent different definitions, we used the types of scale (Table 2) summarized and modified by Wu and Zhao-Liang (2009).

**Table 2.** Scale definitions summarized and modified by Wu and Zhao-Liang (2009), their comments and remarks on these definitions, and our assessment of the level of ambiguity and applicability for each type of scale and their definitions.

Types of spatial scale	Scale definitions	Comments and remarks on the definitions	Level of ambiguity and applicability of the scale definitions
Cartographic scale	The ratio between a particular distance on a map and on the same distance on the ground	A small cartographic scale corresponds to a large geographic scale and may show fewer features or less detail	*****
Geographic scale	The spatial extent of a research area	A large geographic scale study involves a large spatial area and a small geographic scale study only contains a small spatial area	*****
Modelling scale	The dimension at which the data is acquired or derived	In order to better reveal the processes being modeled, the “Modelling scale” should match both the “Observation scale” and the “Operational scale”	**
Operational scale	The dimension of an action at which a certain process is assumed to operate	This dimension depends on the nature of processes being investigated. Variability at smaller scale than the “Modelling scale” may be lost if the “Operational scale” is smaller than “Modelling scale”	**
Policy scale	The dimension scale at which decisions are made or a policy is implemented	In order to draw reliable conclusions, the “Policy scale” should be larger than “Operational scale”	**
Observation scale	The measurement or sampling intervals at which data is measured or sampled	This can refer to the resolution, time interval, spectral range, solid angle or polarization direction	****
Measurement scale	The measurement or sampling intervals at which data is measured or sampled	This can refer to the resolution, time interval, spectral range, solid angle or polarization direction	****

The same authors argued that observation, Modelling, geographic, and cartographic scales are more closely related to remote sensing, whereas policy scale (determined by policymakers), and operational scale (as a natural characteristic of the process) have less to do with remote sensing.

Accordingly, we attempted to evaluate the assumption provided by Wu and Zhao-Liang (2009), using stars to represent the levels of ambiguity and applicability for each scale definition; with one star (*) indicating that the scale definition is very ambiguous and may, therefore, be inapplicable, and with five stars (*****) indicating that the scale definition is very precise and hence likely to be very applicable. Table 2 is subsequently re-evaluated on the basis of the survey results.

## Survey design

The survey was conducted in English and was accessible online through the internet-based Survey Monkey platform. The survey was designed with four main objectives in mind:

- To evaluate the relative importance of spatial and temporal scales
- To evaluate the relative importance of the different types of scale
- To evaluate the ambiguity and applicability of the scale definitions
- To evaluate the relative importance of studying objects and phenomena at multiple spatial scales

### Structure of the survey

**Figure 1.**
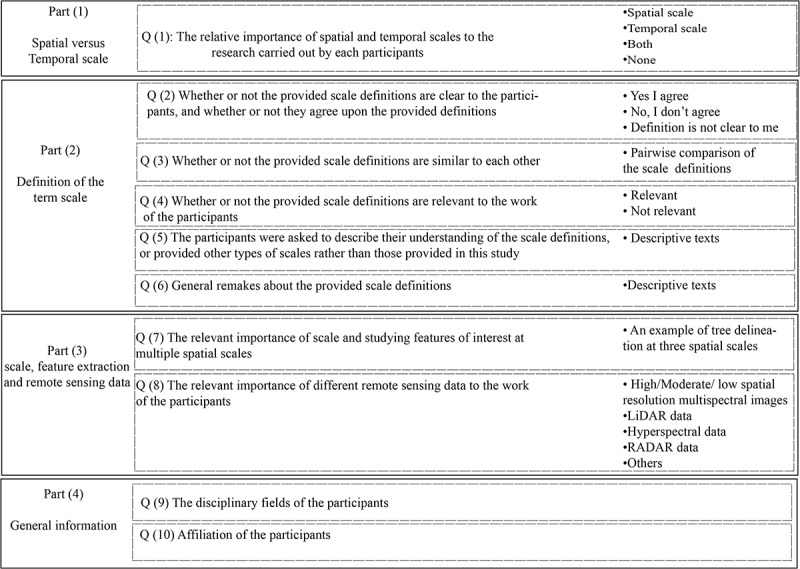
An illustration of the structure of the survey.

The survey comprised 11 main questions of which eight were multiple choice questions, with the rest requiring descriptive answers. Figure 1 shows the overall structure of the questionnaire.

#### Part one: evaluation of the relative importance of temporal and spatial scales

The first question in the survey (Question one) addressed the relative importance of spatial and temporal scales to the research carried out by each participant; it also included an option to indicate that neither type of scale was important.

#### Part two: evaluation of published definitions for different types of scale

The second part of the survey sought to evaluate the ambiguities and/or uncertainties in the scale definitions provided, as well as the relative importance of the different types of scale to the work of the participants. This part comprised five main questions (Question two to Question six), as follows:

Question two was designed to evaluate the ambiguities and/or uncertainties in the definitions provided for different types of scale. The survey sought to evaluate (1) whether or not the scale definitions in Table 2 were clear to the participants, (2) whether or not they agreed with the definitions, and (3) whether or not the definitions were ambiguous.

Question three aimed to discover any similarities that the participants identified between the different scale definitions, based on their own particular work experiences. This was achieved by comparing the different scale definitions with each other in turn.

Question four was designed to assess the relative importance of the different types of scale to the work of the participants, which the participants were asked to determine by choosing either this type of scale is important or not important.

Question five asked the participants to describe the types of scale or definitions of scale other than those provided in the survey, that important to their work.

Question six was a descriptive question, which the participants to present their own comments, suggestions, and general remarks on the scale definitions provided in the survey.

#### Part three: evaluation of the relative importance of scale on studying objects and/or phenomena at multiple spatial scales

Question seven was focused on evaluating the relative importance of scale on studying objects and/or phenomena at multiple spatial scales. For demonstration purposes, an example showing the delineation of trees and tree communities from satellite imagery at three spatial scales (fine, medium and coarse) was presented (Table 3). The participants were asked to determine which of these three spatial scales were the most relevant to their work. The participants were allowed to choose multiple spatial scales.

**Table 3.** Illustration of the hypothetical delineation of three spatial scales from remote sensing imagery: (1) fine spatial scale, showing delineation of individual trees, (2) medium spatial scale, showing the delineation of stands of trees, and (3) coarse spatial scale, showing the delineation of forested areas.

Question seven: Which of the following spatial scales are the most relevant to your work?		
		
Fine spatial scale, for delineation of individual objects (e.g. individual trees)	Medium spatial scale, for delineation of more general objects (e.g. stands of trees)	Coarse spatial scale, for delineation of population or communities (e.g. forested areas

Question eight was designed specifically for those participants which might have remote sensing background, and to evaluate the relevant importance of different types of remote sensing imagery at their work.

#### Part four: information on the survey participants

The fourth part of the survey gathered general information on the participants, including the main field of work and their affiliation of the participants.

## Results

### Analysis of survey results

#### Part 1: evaluation of the relative importance of spatial and/or temporal scales

The results of the relative importance of spatial and/or temporal scales on the work of the participants revealed that the majority of the participants (55%) chose both spatial and temporal scales to be important to their work. The second majority belonged to the importance of spatial scales (45%).

#### Part 2: evaluation of published definitions of scale

Responses to Question two revealed a high level of acceptance (ranging from 60% to 95%) of the presented definitions for different types of scale (Table 4).

**Table 4.** Evaluation by participants of the definitions provided for different types of scale.

Question two: “Do you agree with the definitions provided for the following types of scale?” (n = 142)			
Types of scale	(a) Yes, I agree (%)	(b) No, I don’t agree (%)	(c) Definition is ambiguous (%)
Cartographic scale	95	2	3
Geographic scale	69	19	12
Operational scale	81	4	15
Observation scale	60	17	23
Measurement scale	73	12	14
Modelling scale	86	3	12
Policy scale	73	6	21

According to Table 4, the definition of “Cartographic scale” seemed to be clear to the vast majority of the participants (95%), and this definition was only rejected by a small number of participants (2%). The participants agreed upon the definitions of the “Modelling scale” (86%), “Operational scale” (81%), and the “Policy scale” (73%), and therefore, they were assigned to be less ambiguous. Although the definitions provided for “Observation scale” and “Measurement scale” were the same, the results revealed that the term “Measurement scale” (73%) was better received in comparison to the term “Observation scale“ (60%). The definitions provided for “Observation scale” (60%) and the “Geographic scale” (69%) had the lowest level of agreement and, the definition of “Observation scale” (23%) gained the highest level of ambiguities among the participants.

**Figure 2.**
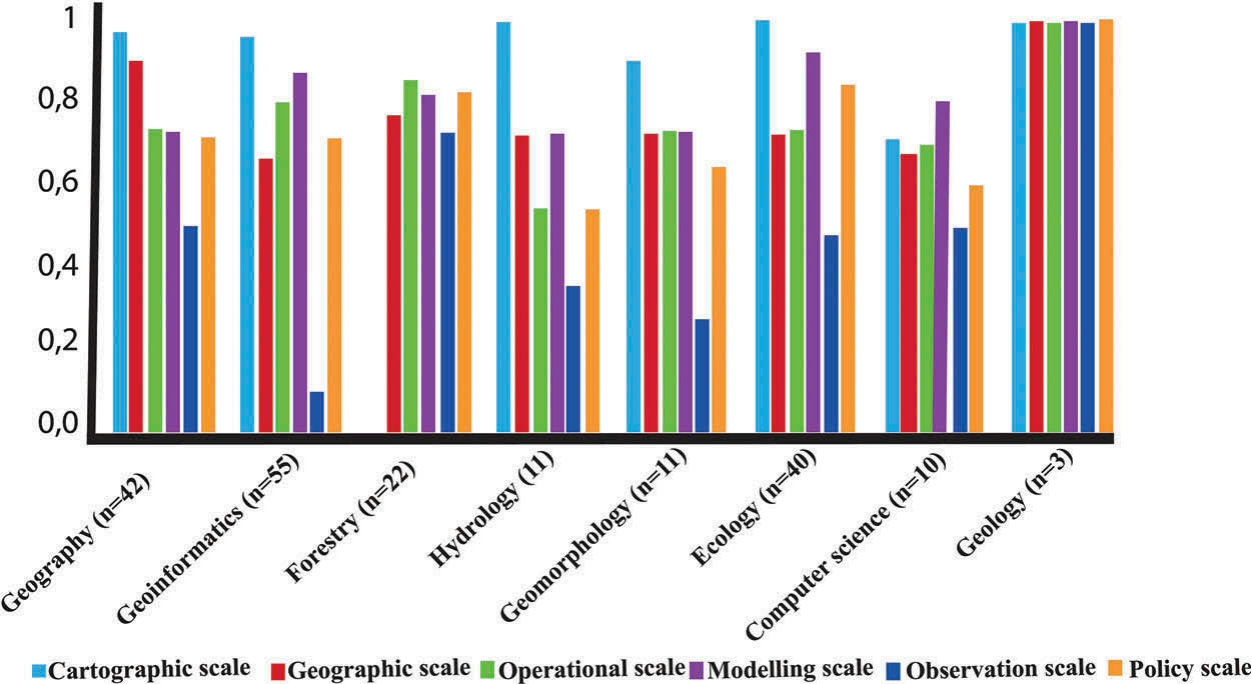
the relationship between the disciplinary fields of the participants and the scale definitions that they found well defined.

In addition, the relationship between the disciplinary fields of the participants and the scale definitions that they found well-defined (Table 4) visualized in Figure 2.

In Question two, participants were able to add their own comments explaining their understanding of the definitions. Most participants commented on the similarities and overlap between the various definitions provided for different types of scale. Some participants said that they had never heard of some of the scale categories (such as, for example, “Policy scale”). Some participants also said that semantics was an important aspect of understanding and applying scale definitions and that, for instance, those who do not have English as their mother-tongue might find the provided scale definitions misleading. Summaries of these comments are provided in the “Discussion” and the “Conclusion” sections.

The next question (Question three) addressed the level of similarities between the definitions for different types of scale through pairwise comparisons of the definitions (Table 5).

**Table 5.** Evaluation of the similarities between the definitions of different types of scale.

Question three: Do you see similarities between the definitions of different types of scale? (n=150)							
	Percentage of participants reporting similarities between two types of scale (%)*						
Types of scale	Cartographic scale	Geographic scale	Operational scale	Observation scale	Measurement scale	Modelling scale	Policy scale
Cartographic scale		15	3	7	12	11	
Geographic scale			16	13	10	11	15
Operational scale				11	13	19	3
Observation scale					37	17	7
Measurement scale						9	12
Modelling scale							11
Policy scale							

*Percentage between each two types of scale was calculated by: dividing number of responds for each pairwise comparison by total number of participants (150), and then the result multiplied by 100.

According to the results of comparing the different scale definitions (Table 5) participants selected “Operational scale” to have the highest level of overlap with other definitions, specifically those for “Modelling scale” (19%), “Geographical scale” (16%), “Policy scale” (12%), and “Observation scale” (11%). The second-high similarities among scale definitions belonged to the definition provided for “Geographic scale” to be similar to those provided for “Operational scale” (16%), “Cartographic scale” (15%), “Observation scales” (13%), and “Modelling scale” (11%). The similarity between two terms “Measurement scale” and the “Observation scale” was recognized by 37% of the participants, although the scale definitions provided for both were the same.

Table 6 presents a summary of the responses to Question four regarding the importance of the different types of scale to the work of the participants.

**Table 6.** The importance of the different types of scale to the work of the participants.

Question four: “How important are the following types of scale to your work?” (n = 150)	
Types of scale	Important (%)
Cartographic scale	86
Geographic scale	86
Operational scale	76
Modelling scale	76
Observation scale	90
Policy scale	58

Five types of scale were considered important by more than 76% of the participants (Cartographic, Geographic, Operational, Modelling, Observation scales) yielded high levels of importance. Policy scale was considered to be less important to (by 58%) to the work of the participants.

**Figure 3.**
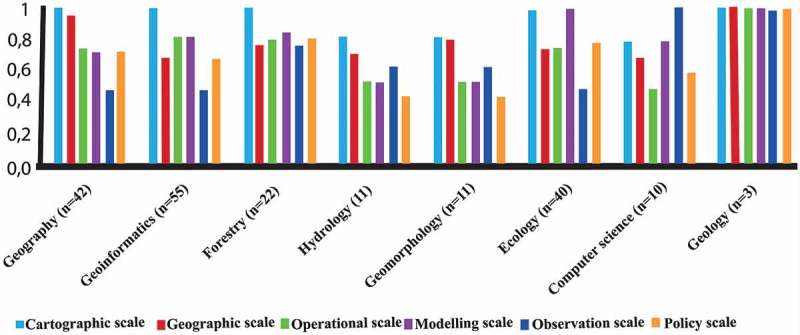
Relationships between relevancy of scale definitions and background domain of the participants.

Figure 3 illustrates relationships between the importance of scale definitions and background domain of the participants. All scale definitions were chosen to be relevant among different geoscience disciplines.

A final free-text question in part two addressed other types of scale or definition, in addition to the types of scale presented in the survey. The majority of participants argued that temporal scales were very important and that they should also be considered. Table 7 shows some of the additional types of scale and definitions suggested by the participants. Only those suggestions that were made by more than one participant have been used.

**Table 7.** Additional types of scale and definitions suggested by participants.

Question five: “Can you think of any other categories of scale, apart from those that were mentioned in the survey?” (n = 94)	
Suggested types of scale:	Definitions provided by the participants:
Temporal scale	The time intervals over which processes influence or change spatial situations
Administrative scale	Refers to administrative levels such as community, county, federal state, national state, …
Stakeholder (or decision-maker) scale	The dimension over which stakeholders or decision makers take their decisions
Ecological scale	The combination of extent and resolution at which research is conducted, which is also assumed to take into account operational scale
Grain size and extent scale	In landscape ecology, they are defined by the grain size (the finest spatial or temporal resolution of the dataset) and either the spatial extent of the study area or the period covered by the observations
Analysis scale	The dimension at which a problem is analysed

#### Part 3: evaluation of the importance of scale on studying objects and/or phenomena

Table 8 shows the results of the importance of scale on the work of the participants. The results revealed that the medium and coarser scales were the most important to the work on the participants (91%, 84%, respectively). The fine scale, and studying individual objects, was relevant to 72% of the participants.

**Table 8.** The most important spatial scales to the work of the participants.

Question seven: “Which of the following spatial scales is the most important to your work?” (n = 95)	
Spatial scale	Important (%)
(1) Individual objects	72
(2) Groups of similar objects/populations	91
(3) Communities	84

**Figure 4.**
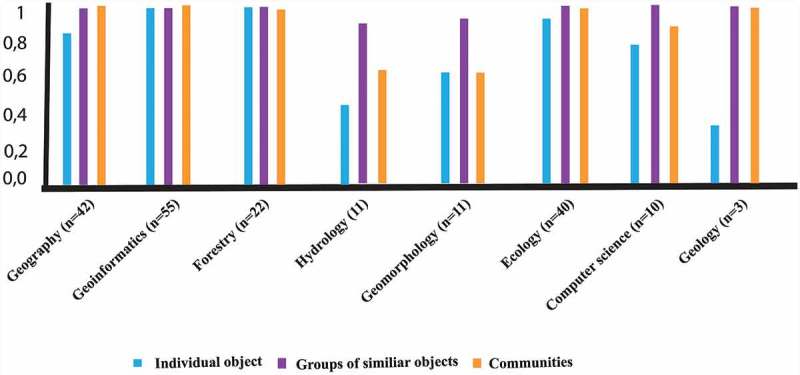
relationships between the relevancy of each three mentioned spatial scales to the background domain of the participants.

Figure 4 illustrates the relationships between the relevancy of each three mentioned spatial scales to the background domain of the participants. Participants with the geoinformatics, forestry and ecology chose all the suggested spatial scales to be relevant to their work. Participants with the hydrology, geomorphology, and computer science chose the second spatial scale (i.e. groups of similar objects or populations) to be relevant to their work. And participants with the geology background chose the second and the third spatial scales to be relevant to their work.

We also examined either each participant chose more than one scale to be important in their work. Six possible scenarios were foreseen as shown in Table 9. The majority of the participants preferred to study objects and phenomena at more than one spatial scale, either at all three of the predefined spatial scales (60%) or at two of the three spatial scales (29%). Only a minor group of participants (8%) indicated only one scale to be relevant to their work.

**Table 9.** The importance of studying objects at multiple spatial scales.

Spatial scales	Important (%)
All three spatial scales	60
A combination of spatial scales for delineating individual objects (e.g. individual trees), and groups of similar objects (e.g. tree stands)	8
A combination of spatial scales for delineating groups of similar objects (e.g. tree stands), and communities (e.g. forested areas)	23
A combination of spatial scales for delineating individual objects (individual trees), and communities (forested areas)	0
Only one of the three spatial scales	9

The last question of the survey aimed to determine the type of remote sensing imagery most widely used by the participants (Table 10). When presented with a choice between radar imagery and optical imagery, 90% of the participants chose optical imagery as the most important type of remote sensing imagery for their work. Subsequent comparisons between different types of optical imagery showed that “very high/high resolution imagery” was the most important to the work of 32% of the participants, with “multispectral medium to low-resolution imagery” being most important to 23% of the participants, “LiDAR datasets” to 18% of the participants, and “hyperspectral imagery” to 17% of the participants.

**Table 10.** Evaluation of the importance of different types of remote sensing imagery to the work of the participants (multiple choices).

Question nine: “Which types of remote sensing imagery are the most important to your work? (n = 84)		
Types of remote sensing data	Relevant to their works (number of participants out of 84)	Relevant to their works (%)
Very high/high resolution imagery	72	86
Multispectral medium/low resolution imagery	52	68
Hyperspectral imagery	40	60
LiDAR datasets	37	54
RADAR imagery	23	38

### Information on the survey participants

**Figure 5.**
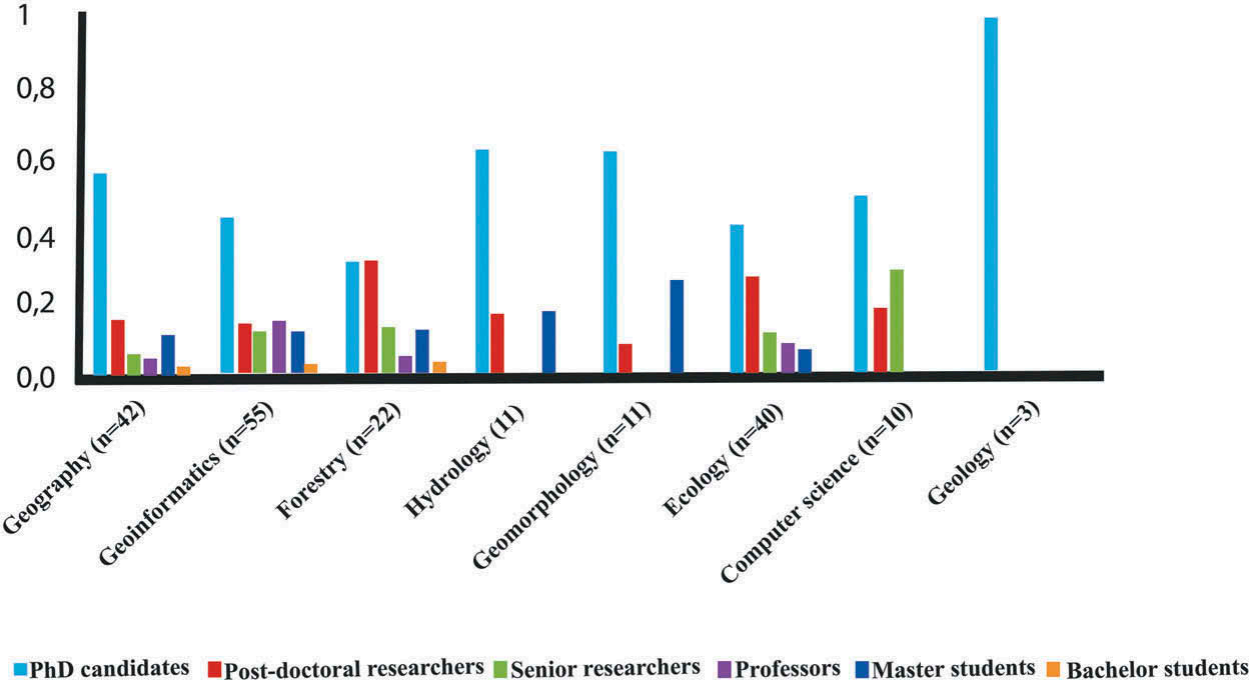
Proportion of participants from different disciplinary backgrounds, also showing their career status.

A total of 150 participants contributed to this survey. We aimed for balanced sample distribution for three major groups, including (1) PhD candidates, (2) researchers (senior and postdoctoral researchers and professors), (3) graduate and undergraduate students (i.e. master and bachelor students). The participants came from a variety of disciplines: 26% from “Geoinformatics and Remote sensing”, 22% from “Geography and Cartography”, 10% from ”]Ecology”, and 11” from “Landscape ecology”, 11% from “Forestry and Agriculture”, 10% from “Geomorphology, Geology, Geodesy, Limnology, and Soil science”, 5% from “Hydrology, Meteorology” and, lastly, 5% from “Computer science”. Most of the respondents (52%) were Ph.D. students, followed by post-doctoral researchers (18%), professors (10%), master’s students (10%), senior researchers (8%) and bachelor’s students (2%). Figure 5 illustrates the disciplinary background and career status of the participants summarized in the eight groups.

**Figure 6.**
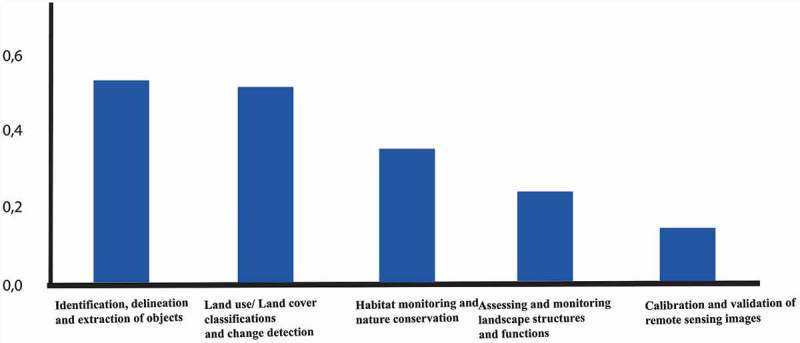
Main fields of application for which remote sensing imagery was used by the participants.

The main fields of applications for which remote sensing imagery was used by the participants. The applications were broken down into five particular tasks (Figure 6). The two tasks most commonly selected were “Identification, delineation, and extraction of objects/features from remote sensing imagery” and “Land use/land cover classifications and change detection”.

## Discussion

### Evaluation of the importance of spatial and temporal scales

Warner et al. (2009) suggested that, in order to obtain reliable results, the scale of analysis should match the scale at which the processes under investigation are assumed to operate. The first objective of this research was to evaluate the relative importance of spatial scales to the work of researchers. Turner (1989) described the scale dependency of patterns and processes, claiming that spatial and temporal scales need to be clearly defined. The results of our survey clearly confirmed the importance of both spatial and temporal scales to the work of participants, with 98% of the participants stating that either spatial or temporal scales (or both) were important to their work.

### Evaluation of scale definitions

Scale is a critical factor when studying patterns, processes, and changes in physical entities, and has been widely recognized as a key consideration when studying human-environment interactions (Levin, 1992). However, certain concepts of scale remain unclear and ambiguous (Marceau, 1999). Wu and Li stated that “Clarification of key concepts is the first step towards a science of scale” (Wu & Harbin, 2006, p. 13). The list of scale definitions summarized and provided by Wu and Zhao-Liang (2009) was used in the survey to investigate the current state of different types of scales.

**Figure 7.**
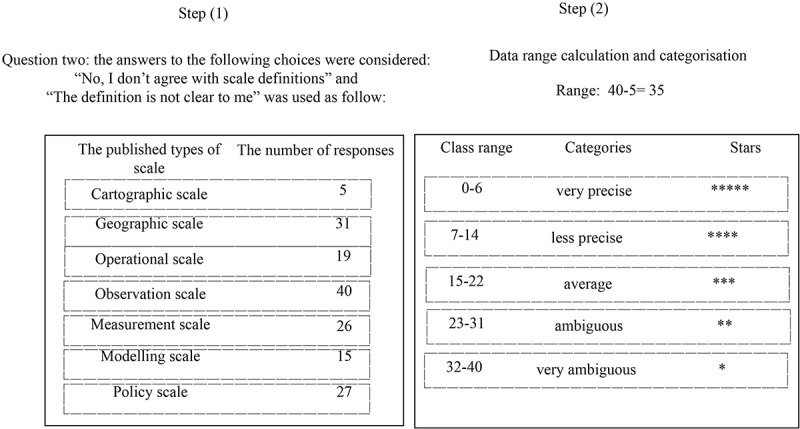
Evaluation of ambiguities in the scale definitions, using the responses to question two of the survey.

It was hypothesized in section 2.4 that some of the scale definitions are ambiguous and hence less applicable. According to the responses to Question two of the survey (presented in Table 4), the summation of the two categories: “no, I don’t agree” and “the scale definition is not clear to me” was used to evaluate our assumption presented in Table 2. The workflow to evaluate our assumption is shown in Figure 7. The assumption was, those scale definitions that gained more responses for the choices “no, I don’t agree” and “the scale definition is not clear to me” are more ambiguous, and vice versa.

According to Figure 6, the following classes can be derived:

- Cartographic scale (value: 5), class range: 0–6, very precise, five stars (*****)
- Geographic scale (value 31), class range: 23–31, Ambiguous, two stars (**)
- Operational scale (value 19), class range: 15–22, Average, three starts (***)
- Observation scale (value 40), class range: 33–40, very ambiguous, one star (*)
- Modelling scale (value 15), class range: 15–22, Average, three stars (***)
- Policy scale (value 27), class range: 23–31, Ambiguous, two stars (**)

Table 11 shows that the authors’ initial evaluations for “Cartographic scale” (five stars), and “Policy scale” (two stars) remained unchanged following the analysis of the survey results. The main differences were for the definitions of “Geographic scale” and “Observation (measurement) scale”. Both terms were downgraded following analysis of the survey results, although this may have been because the participants came from a variety of spatial disciplines. “Geographic scale” and “Observation (measurement) scale” were the most important types of scale to their work (see Table 6), and there is, therefore, a need for more explicit definitions of these two types of scale. “Modelling scale” and “Operational scale” were initially assigned three stars but this was reduced to two stars following the analysis of responses to the survey, which suggests that the authors’ initial evaluations regarding the ambiguities in the definitions of these two types of scale were more or less correct (Table 11).

**Table 11.** Scale definitions adopted and modified by Wu and Zhao-Liang (2009), together with assessments of their level of ambiguity by the authors and from the survey results (based on responses to question two).

Types of scale	Scale definitions	Level of unambiguity (applicability) assessed by the authors	Evaluation of the initial authors suggestion for ambiguities of scale, according to participants responds
Cartographic scale	The ratio between a particular distance on a map and on the same distance on the ground	*****	*****
Geographic scale	The spatial extent of a research area	*****	**
Modelling scale	The dimension at which the data is acquired or derived	**	***
Operational scale	The dimension of an action at which a certain process is assumed to operate	**	***
Policy scale	The dimension scale at which decisions are made or a policy is implemented	**	**
Observation scale	The measurement or sampling intervals at which data is measured or sampled	****	*
Measurement scale	The measurement or sampling intervals at which data is measured or sampled	****	**

Analysis of the survey results showed that more than 60% of the participants agreed with the scale definitions provided and indicated which types of scale were important to their work. However, some participants either did not agree with the scale definitions provided or found them ambiguous. In general, there are three views regarding scale definitions; (1) some participants claimed for clearer definitions of scale, (2) delimitation of scale definitions according to the field of study, and (3) a scale definition shall be more “relaxed”, meaning the scale definitions shall be broad enough to cover a broad range of disciplines. The comments made by participants on the provided types of scale used in the survey are summarized as follows.

#### Cartographic scale

“Cartographic scale” was the type of scale most generally accepted by the participants; a cartographic scale is a mathematical expression that indicates the relationship between a modelled area and the real-world extent of the area. It was argued by some participants that “Cartographic scale” could be considered to be part of the “Modelling scale”, as both definitions are used to define models. It was also argued that “Cartographic scale” was a purely geometric spatial concept that, together with the resolution of datasets (i.e. the “Observation scale”), limits the ability to identify geographic objects. However, although these two types of scale are similar and comparable in terms of object identification, differences remain as “Cartographic scale” refers to map representations for display purposes and is expressed as a ratio, while “Observation scale” refers to the acquisition of data and is expressed as spatial or temporal types of scale.

#### Geographic scale

“Geographic scale” is most widely understood in terms of dimensional characteristics, such as the extent (or dimensions) of a study area. However, the extent of a research area is entirely subjective and dependent on the researcher’s choice of the spatial extent of the research area. Some participants also suggested that the definition provided for “Geographic scale” was too general to be useful and therefore needed to be redefined, for example, by including some indication of what is regarded to be a large scale and what is regarded to be a small scale. It was suggested that the definition should include an indication of what is mean by local, regional, and global scales, and how they can be distinguished. For some of the participants, it seemed to be more feasible to use extent and resolution than “Geographic scale”. Although the definition of “Geographic scale” indicates a dimension and the definition of “Cartographic scale” is a unitless ratio some participants, however, claimed that the definition of “Geographic scale” overlapped with that of “Cartographic scale”.

Some comments addressed overlaps between the definitions of “Geographic scale” and “Operational scale”, and between those of “Observation scale”, “Modelling scale”, and “Cartographic scale”. Some of the survey participants felt that the purpose of all these definitions was to identify particular processes and spatial and temporal extents over which these processes operate. A small number of participants also stated that the “Geographic scale” in the sense of the extent of the research area (e.g. local, regional, or country-wide extent, etc.) should be referred to as a “level” rather than a “scale”. However, this is not in agreement with the mainstream scientific literature, as discussed in the introduction.

#### Operational scale

Some participants stated that the definition of “Operational scale” was too general as it may not consider the relationship between processes and the respective geographical extent of the research area. A few participants also asserted that it defines a measurement (for example, measurement of length, volume, etc.) as do all of the other definitions except “Cartographic scale” (and possibly “Modelling scale”), which is a ratio rather than a dimension. A definition should consequently consider the fact that processes and interactions rarely have distinct boundaries, and that they are likely to function over a number of spatial and temporal scales. In addition, temporal scales are an important aspect of the study of processes and should therefore also be considered in the definition. Finally, some of the participants claimed that they had never heard of “Operational scales”.

#### Observation and/or measurement scale

In the survey participants selected the definition of “Observation scale” as the most ambiguous of all the definitions provided (Table 4). When referring to measurement or sampling intervals, the term “Measurement scale” thought to be more appropriate than “Observation scale”. Some participants also argued that “Observation scale” and “Modelling scale” were interdependent since the modelled objects cannot be smaller than individual measurements, which means that the “Observation scale” places a limit on the “Modelling scale”. According to the literature (Table 2), we provided the same meaning for the “Observation scale” and the “measurement scale”; it was argued by the participants that there must be clearer definitions for these two terms.

#### Modelling scale

The definitions of “Modelling scale” and “Cartographic scale” were found to be the most acceptable (Table 4). As mentioned previously, “Cartographic scale” can be considered to be a special case of “Modelling scale” in which the model is described by a mathematical expression. It was also argued by some participants that there is a connection between “Modelling scale” and “Policy scale” since policy decisions are often made on the basis of outputs from models. Once again, some of the participants claimed that they had never heard of “Modelling scales”.

#### Policy scale

Many of the participants felt that the definition provided for “Policy scale” was very general (and possibly too general), and that is too broad a concept. The majority of the participants that criticized the definition of “Policy scale” thought that this should be referred to as a “Policy level” rather than “Policy scale”, reflecting the administrative levels (or territorial boundaries) at which decisions are made (e.g. local, regional, or national levels). As with some of the other definitions, some participants claimed that they had never heard of “Policy scale”, but it is important to have a clear definition of the “Policy scale”.

### Evaluation of the importance of scale on studying objects and/or phenomena

The spatial and spectral resolutions of remote sensing imagery have improved dramatically in recent years, as has the range of sensors. Sensors today deliver data with a spatial resolution that ranges from high resolution (e.g. less than half meter for WorldView satellite) to medium or coarse resolution (e.g. the average of 10 m for Sentinel-1 and −2 or 30 m spatial resolution for Landsat series data or 250 m and 500 m resolutions for MODIS data). This range of data, together with easier access to the data and the wide availability of different types of imagery, has all combined to substantially increase our ability to study the earth’s surface at different scales. In recent decades theories have been defined to measure, study, and predict patterns and processes at different spatial scales (Allen & Starr, 1982; Burnett & Blaschke, 2003; Wu, 1999). The results from our survey have confirmed the importance of studying patterns at multiple spatial scales, with the majority of participants (60%) indicating that it was important to study objects and phenomena at all three of the predefined spatial scales. The second largest group of participants indicated that it was important to study objects or phenomena at two of the three spatial scales. In the survey example, most participants focused on studying objects and phenomena at spatial scales that were representative of groups of objects (for instance populations or communities), rather than individual objects.

## Conclusions

This study has provided a review of published definitions for different types of the scale used in spatial disciplines. We initially assumed that some of the different types of scale and their definitions might be ambiguous to researchers, and would, therefore, be less applicable. We, therefore, designed a survey based on definitions of different types of scale provided by Wu and Zhao-Liang (2009). We aimed for a balanced sample distribution and interviewed a total of 150 participants. Although the number of participants was just sufficient for statistical analysis, the range of disciplinary backgrounds is a benefit. The results revealed that participants considered all types of scale to be relevant to their work, and the definitions of the different types of scale were also found to be generally acceptable. The majority of the participants agreed upon the definitions provided for “Cartographic scale” (95%), “Modelling scale” (86%), and “Operational scale” (81%). The definitions provided for “Observation scale” (60%), “Geographic scale” (69%) and “Measurement scale” (73%) were still well recognized. The participants showed relatively little knowledge and acceptance for “Observation scale” (23%) and “Policy scale” (21%). The results showed that the more cartography related types of scale are relatively well known while the more abstract dimensions are less known and are most ambiguous. Based on graphical examples, participants were asked which spatial scales are most relevant for their work. Surprisingly, composite objects such as a forest stand were most relevant followed by individual objects such as single trees and, lastly, more generalized categorizes or meta-objects such as “forested area”.

We have drawn the following conclusions that will help to clarify the different types of scale in regard to their practical use:

- The English language has become accepted by the scientific community as the common language of communication; concepts and definitions are either stated in English or translated into English. However, for many researchers, English is only their second language, and some participants in the survey indicated that they consequently had trouble answering some questions because of language problems. The comments clearly emphasizing on the importance of semantic for better describing and conceptualization of the different definitions of scale. This conclusion is a direct consequence of the structure of the population answering the survey.
- Scale definitions are often ambiguous, even within a given discipline. Interdisciplinary use can also result in confusion. For instance, researchers from different disciplines may have different understandings of what is meant by types of “scale” and definitions used in this study. The researchers that participated in this study came from a variety of disciplines, with 76% describing their field of study as multidisciplinary and just 24% indicating only one discipline as their major scientific field. Geoinformatics was a common field of study, and it has connotations of a multidisciplinary field of study (Blaschke & Merschdorf, 2014). We conclude that any scale definition should not be restricted to a specific discipline but should be suitable for multidisciplinary or interdisciplinary use. There is also a need to establishing a conceptual structure to represent the semantic correspondence among scale definitions to resolve ambiguities associated with each types of scale, and different scale definitions.
- Although a plethora of scale definitions exist (especially in geography and ecology) this study has confirmed that “Cartographic scale” is the only type of scale that is acceptable in all fields and all possible usages.
- All of the scale definitions investigated in this study were intended to be reliable and to be transferable between different fields of research. However, this appears to be particularly difficult when investigating human-environment interactions and patterns, and their underlying processes. Virtually every study may unfortunately, therefore, need to include a clear statement of what is understood by the term “scale” or by the particular types of scale referred to the study. This, however, need not involve developing new definitions but can start from existing definitions, such as those provided by Wu and Zhao-Liang (2009).
- This study has been able to verify the assumption of the importance of studying objects and phenomena at multiple spatial scales, which was clearly confirmed by the participants. However, the question of how this can best be achieved is clearly beyond the scope of this article. There exists an increasing body of literature on the importance of studying features and phenomena on multi-scale analysis, and various conceptual frameworks may already exist to partially guide such analyses (Caldwell, Matson, Wessman, & Gamon, 1993; King, 1991; Levin, 1992; Levine, 2000; Turner, 1989; Wiens, 1989; Wu, 1999; Wu et al., 2006; Wu & Qi., 2000). We refer to Levin (1992), that there is no single “correct” scale for describing ecosystems, rather we shall accompany our attention to the interaction among patterns and processes on different scales. We believe that in particular object-based image analysis (OBIA) (Blaschke, 2010; Blaschke & Merschdorf, 2014; Blaschke & Piralilou, 2018; Burnett & Blaschke, 2003; Chen, Weng, Hay, & Yinan, 2018; Georganos et al., 2018; Josselin & Louvet, 2019; Tiede, 2014), can serve as a methodology to generating a structural hierarchy at multiple scales, in particular for the image processing domain.
